# 

**Published:** 1915-04-24

**Authors:** 


### CONTENTS.

| | PAGE |
|---|---|
| **The Call to the Medical Profession** ... ... | 77 |
| **The Army Pharmaceutical Service** ... ... ... | 78 |
| **Hospital and Institutional News** ... ... ... | 79 |
| The New Red Cross Commission—Death of a Great Chairman—Women as Hospital Porters—The Difficulties of Sectional Representation—The Loss of Subscribers through Enlistment—The Local Press and the Monthly Meeting—A Supporter of Hospitals in South Wales—New Chairman of Wood Green Hospital—Workhouse Medical Officer and Fees for Operation—An Infirmary Book-keeping Case—Medical Officers and Clerical Work—St. George's Hospital Wins its Case—Smoking in Hospitals—Dental Treatment for Tuberculous Cases—War Office and the Metropolitan Asylums Board—Wounded at Race Meetings—The Need for a Minister of Public Health—Death of a Mental Superintendent—Abandoning the Consumption Crusade—What Children Can Do—Successful Claim for Wrong Dispensing—This Week's Drug Market—County Asylums as Military Hospitals—To Our Readers. | |
| **Cerebro-Spinal Meningitis** ... ... ... ... | 85 |
| Its Infectivity and the Serum Treatment. | |
| **The Standard Opaque Meal for X-Ray Examination** ... ... ... ... ... | 86 |
| The Cheapness of Barium Sulphate. | |
| **Chronic Collargol Poisoning** ... ... ... | 86 |
| **Problems in Administrative Medicine** ... ... | 87 |
| Medical Reports on Hospital Patients. | |
| **Round the World** ... ... ... ... ... | 88 |
| Fellow-Workers and the Roll of Honour. | |
| **The Report of the Local Government Board** ... | 89 |
| A Review of its Poor-Law Medical Work. | |
| **St. Bartholomew's Hospital: Reports on Patients** ... ... ... ... ... ... | 90 |
| Text of the Rules for the Medical Staff. | |

[See over.]

### JAMES SLATER & CO. (Engineers) LTD.

**50 & 51 WELLS STREET, W.**

**HEATING, HOT WATER SUPPLY, VENTILATION, ETC.**

We make a great speciality of this, and are at present engaged upon the entire installation at the new King's College Hospital, including central steam boiler plant, etc., etc.

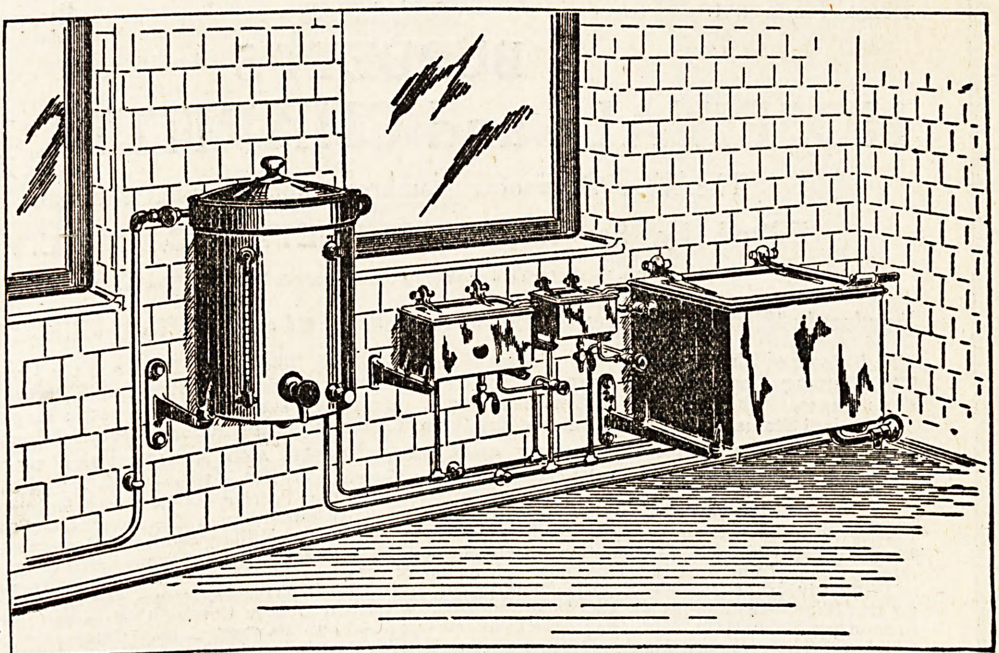

STERILIZERS, as supplied to London Hospital, Middlesex Hospital, Manchester Royal Infirmary, etc.

### CONTENTS—(*continued*).

| | PAGE |
|---|---|
| **Building a Hospital in India** ... ... ... | 91 |
| II.—The Story of the Karnal District Hospital. By Major C. H. Buck, I.A., Deputy Commissioner. | |
| **King Edward's Hospital Fund for London** .. | 93 |
| Annual Meeting of Governors and General Council. | |
| The King's Tribute to the late Lord Rothschild. | |
| The New Treasurer and Committeemen. | |
| The Council's Report. | |
| The Speaker's Address. | |
| **London Panel Committee** ... ... ... ... | 96 |
| **The Editor's Letter-Box** ... ... ... ... | 96 |
| Khaki, Rank, and Recognition for Dispensers in Military Hospitals. | |
| **Medical Appointments** ... ... ... ... | 98 |
| **Gifts and Bequests** ... ... ... ... ... | 98 |
| **Personalia** ... ... ... ... ... ... ... | 98 |
| **The Doyen of the Medical Profession at Cardiff** ... ... ... ... ... ... | 98 |
| **The Book Market** ... ... ... ... ... | 98 |
| **Institutional Needs** ... ... ... ... ... | 98 |
| **The Institutional Worker** ... ... (Suppl.) | 1 |
| IV.—The Almoner (*continued*). | |
| Questions for April. | |
| Questions Invited. | |
| Enquiries and Answers. | |
| The Sick and in Need. | |
| Practical Matters. | |
| War Matters. | |
| Miscellaneous. | |
| Acknowledgments. | |

